# Assessing the Quality of Artificial Intelligence (AI)-Generated Patient Education for Gender-Affirming Top Surgery Using the Modified Ensuring Quality Information for Patients (mEQIP) Tool

**DOI:** 10.7759/cureus.106607

**Published:** 2026-04-07

**Authors:** Adrian O Campos, Mohammed Almeflehi, Sean Kim, Daniel Freet

**Affiliations:** 1 Plastic Reconstructive Surgery, University of Iowa Hospitals and Clinics, Iowa City, USA

**Keywords:** artificial intelligence (ai), gender-affirming surgery, large language model, patient education, top surgery

## Abstract

Background: Large language models, such as OpenAI's ChatGPT, have the potential to revolutionize patient education. These platforms allow for a vast collection, analysis, and organization of information largely unavailable to online users. Within medicine, these tools could help complement physicians in better educating patients on complex and routine medical information. Currently, limited literature exists on the reliability of such tools to provide high-quality information to patients inquiring about gender-affirming top surgery. Therefore, this study aimed to evaluate ChatGPT's performance when generating patient-level information on gender-affirming top surgery compared with the current online information provided by the American Society of Plastic Surgery (ASPS) using the Modified Ensuring Quality Information for Patients (mEQIP) tool.

Methods: ChatGPT-4-generated patient-level education on transmasculine and transfeminine gender-affirming top surgery was compared against current online content provided by the ASPS. ChatGPT-4 patient content was generated by individually formatting standardized mEQIP content items to incorporate the topic of gender-affirming top surgery into ChatGPT-4, with responses recorded for each item. Four experts in gender-affirming top surgery independently rated both sources using a 36-item mEQIP tool. Paired t-tests comparing overall and content-specific mEQIP scores of the ChatGPT-4 and ASPS material were then estimated to measure the quality of the content. The effect size between the two groups was evaluated using Cohen's d. Lastly, Cronbach’s alpha and ICC (Intraclass Correlation Coefficient) were calculated to measure internal consistency among raters and interrater agreement.

Results: When analyzing ChatGPT-4 and ASPS patient material, paired t-tests showed a statistically significant increase in overall mEQIP scores for ChatGPT with a mean difference of 7.50 (CI 6.75-8.25; p<0.001). For the mEQIP content-specific scores, a paired t-test revealed a similarly significant increase in ChatGPT scores with a mean difference of 9.75 (CI 9.26-10.24; p<0.001). When evaluating the effect size, a paired Cohen's d value of 13.00 was calculated, demonstrating a statistically significant difference in magnitude between the two groups. To measure internal consistency among raters and interrater agreement, an ICC and Cronbach's alpha were calculated for both ASPS and ChatGPT. The ASPS-mEQIP showed good internal consistency and excellent interrater reliability (ICC=0.89, Cronbach's α=0.84), while ChatGPT-mEQIP showed excellent internal consistency and excellent interrater reliability (ICC=0.96, Cronbach's α=0.96).

Conclusion: These results demonstrate that ChatGPT-4-generated patient education on gender-affirming top surgery exceeded the current ASPS online content in both overall and content-specific scores, as measured by the mEQIP tool. ChatGPT achieved significantly higher scores across both domains with large effect sizes, and raters demonstrated excellent internal consistency and excellent interrater reliability. Going forward, commonly accessible artificial Intelligences (AIs), such as ChatGPT, may serve as a valuable complement to patient education and shared decision-making within plastic and reconstructive surgery, though future studies are warranted to evaluate freely generated responses to better reflect current AI use.

## Introduction

Since the advent of the Internet, patients have utilized internet-based resources to gather medical information. However, despite access to large swathes of health information, patients' ability to understand such material varies widely. According to the American Medical Association (AMA), the readability of patient educational resources should be no greater than a sixth-grade reading level [1]. Prior studies have observed age, education, ethnicity, and income as consistent correlates of health literacy, while also estimating that nearly one-quarter of patients have low health literacy and an additional fifth of subjects have marginal health literacy [2]. Additionally, as more health-related content becomes accessible online, patient utilization of online literature has begun to influence patient education and decision-making [3]. With continued online advancements and internet accessibility, the potential for misunderstanding between surgeons and patients may heighten, especially within plastic surgery. Notably, plastic surgery resources often exceed the recommended reading level for patients, posing a greater need to consider patient comprehension of surgical benefits, operative outcomes, and surgical risks [4]. Thus, the need for comprehensible, high-quality patient-level health information, particularly in plastic surgery, is critical.

To augment these challenges, rapid integration of advanced internet-based tools such as artificial intelligence (AI) has begun. The most popular AI tool, OpenAI's ChatGPT, is designed with an easy-to-use interface that allows for conversational language between the user and the platform [5]. ChatGPT's ability to collect, organize, and analyze large pools of information from online sources allows for automation of tasks and communication of complex processes to users [6]. Beyond content retrieval, ChatGPT possesses the ability to summarize numerous medical concepts, generate personalized educational material, and reframe information at varying levels of literacy. Thus, ChatGPT may be an appealing resource for patients seeking to understand medical procedures. Therefore, as AI tools further integrate across society, health care professionals may anticipate patient self-education with information generated by AI.

Recently, ChatGPT's performance across several domains of medicine has been explored. Prior work has evaluated the tool's ability to provide patient-level education and answer health-related questions in several specialties, including plastic surgery [7-19]. For patients desiring gender-affirming top surgery, reference to online educational materials from the American Society of Plastic Surgeons (ASPS) is common [20,21]. However, there is a paucity of literature evaluating the accuracy of ChatGPT's ability to educate patients regarding the intricacies of gender-affirming top surgery. Therefore, the aim of this study is to evaluate ChatGPT's ability to provide high-quality patient-level information on gender-affirming top surgery utilizing the Modified Ensuring Quality Information for Patients (mEQIP) tool and directly compare it with validated online health information provided by the ASPS [22].

## Materials and methods

This is a single-institution study completed by the Plastic and Reconstructive Surgery Department at the University of Iowa with Institutional Review Board exemption. OpenAI's ChatGPT-4 model was used to generate responses in comparison with the ASPS patient medical education resources on gender-affirming transmasculine and transfeminine top surgery [20,21]. The quality of ChatGPT-generated responses was evaluated using the mEQIP tool, a validated quality assessment scoring system for health care communication toward patients [22].

**Figure 1:**
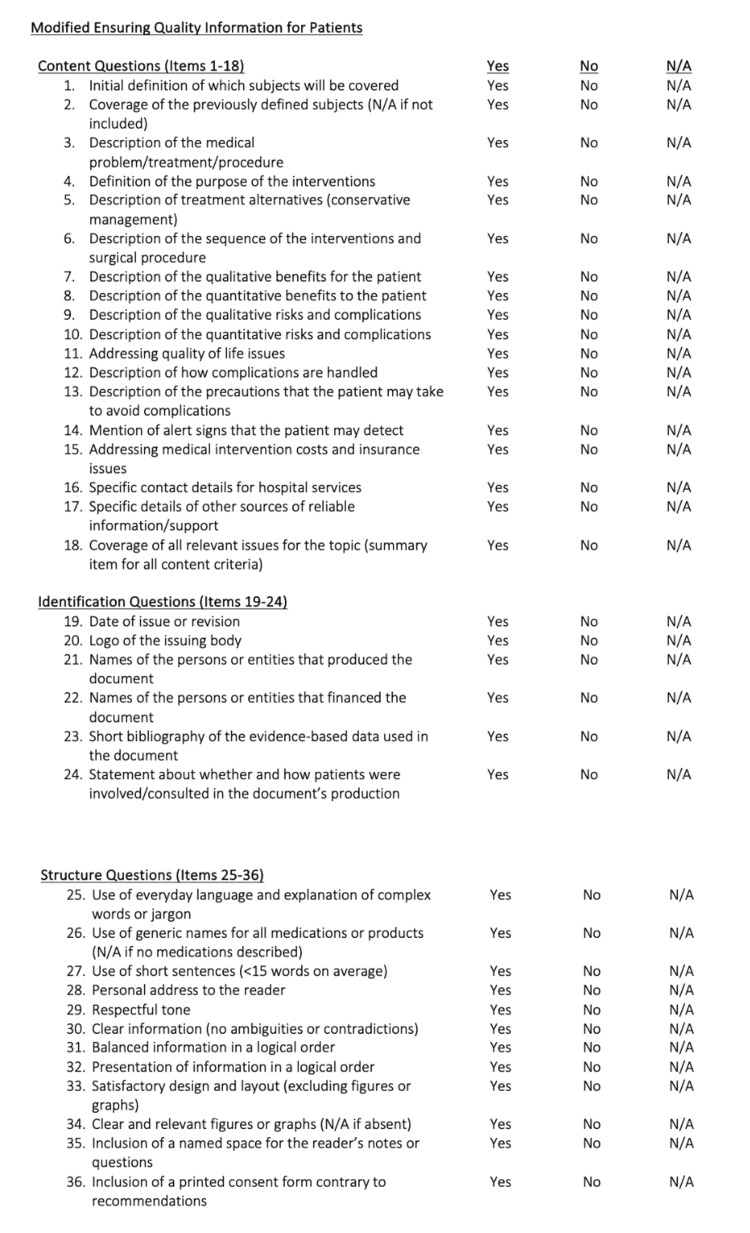
Modified Ensuring Quality Information for Patients Reproduced with permission from Moult B, Franck LS, Brady H. Ensuring Quality Information for Patients: development and preliminary validation of a new instrument to improve the quality of written health care information. Health Expect. 2004;7(2):165-175. Copyright John Wiley & Sons.

**Figure 2:**
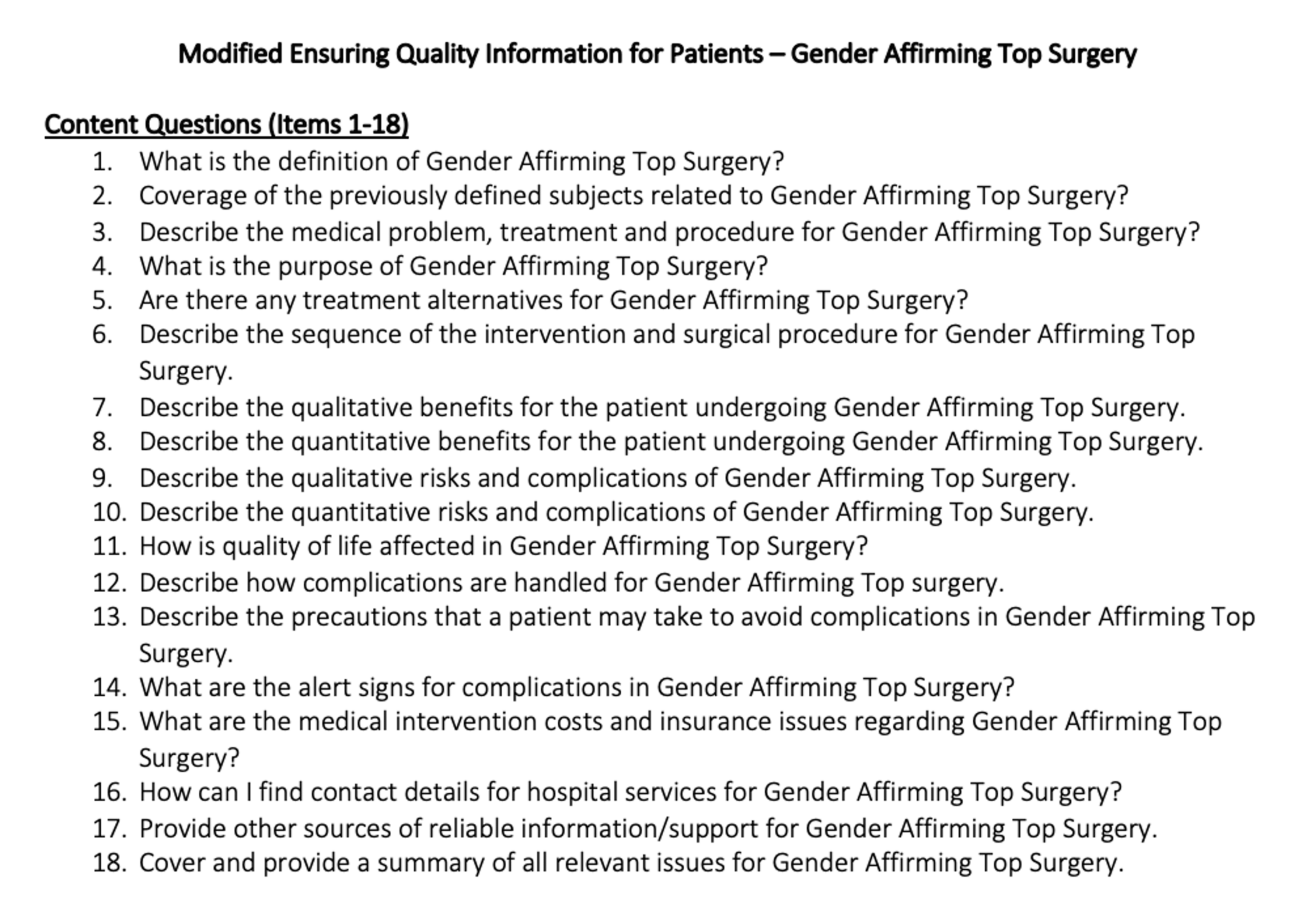
Modified Ensuring Quality Information for Patients (content questions only) - gender-affirming top surgery Adapted with permission from Moult B, Franck LS, Brady H. Ensuring Quality Information for Patients: development and preliminary validation of a new instrument to improve the quality of written health care information. Health Expect. 2004;7(2):165-175. Copyright John Wiley & Sons.

The mEQIP assessment consisted of three domains (Content, Identification, and Structure) and encompassed a total of 36 standardized items (Figure 1) [22]. The standardized mEQIP items related to "Content" were then formatted to incorporate the topic of gender-affirming top surgery (Figure 2), and each item was entered into ChatGPT. ChatGPT-generated responses were recorded and assessed using mEQIP, with each response awarded one point per item for correct and complete answers, zero points per item for incorrect, incomplete, or contradictory answers, or for nonapplicable items. All answers were independently assessed. The resources available through ASPS for gender-affirming top surgery (transmasculine and transfeminine) were also assessed using the mEQIP standardized items with identical application of the point system [20-22]. A total of four raters, considered experts in gender-affirming top surgery, then evaluated the ASPS and ChatGPT-generated gender-affirming top surgery materials using the mEQIP tool.

Paired t-tests were performed to compare ChatGPT and ASPS overall mEQIP scores and "Content"-related mEQIP scores (1-18). The effect size was calculated using Cohen's d value to evaluate the magnitude of the difference between the two groups. In addition, Cronbach's alpha and ICC (Intraclass Correlation Coefficient) were calculated to measure internal consistency among raters and interrater agreement. A statistical significance level of 0.05 was used, along with a 95% CI. IBM SPSS version 22 (IBM Corp, Armonk, NY) was used for all statistical analyses.

## Results

When analyzing ChatGPT-4's and ASPS's patient educational materials, paired t-tests showed a statistically significant increase in overall mEQIP scores for ChatGPT, with a mean difference of 7.50 (CI 6.75, 8.25; p=0.000125) (Table 1). The narrow confidence interval further indicates that the raters' evaluations were highly consistent in distinguishing between the two groups. When evaluating the effect size, a Cohen's d (paired) value of 13.00 was calculated, demonstrating a significant statistical difference in magnitude between the two groups. This appears unrealistically large because of the small sample size, and the differences in scores are almost identical.

**Table 1:** Paired t-test between overall mEQIP scores (#1-36) for ASPS and ChatGPT mEQIP, Modified Ensuring Quality Information for Patients; ASPS, American Society of Plastic Surgery

Characteristics		ASPS*	ChatGPT*	p
mEQIP		18.5	26	0.000125^‡ ^
*Average mEQIP score from 4 raters				
^‡^Statistically significant				

When solely evaluating the "Content" mEQIP scores between the two groups, a paired t-test revealed a statistically significant increase, with ChatGPT receiving higher scores compared with the ASPS materials and a mean difference of 9.75 (CI 9.26, 10.24; p=0.000037) (Table 2).

**Table 2:** Paired t-test between "Content" mEQIP scores (#1-18) mEQIP, Modified Ensuring Quality Information for Patients; ASPS, American Society of Plastic Surgery

Characteristics		ASPS*	ChatGPT*	p
mEQIP		7.25	17	0.000037^‡^
*Average mEQIP score from 4 raters				
^‡^Statistically significant				

The average rater score for each of the "Content"-related items of mEQIP (1-18) was 0.43 for ASPS and 0.94 for ChatGPT (Table 3). These results show that ChatGPT increased the average rater score for "Content"-related items compared with the ASPS content. To measure internal consistency among raters and interrater agreement, an ICC and Cronbach's alpha were calculated for both ASPS and ChatGPT. The ASPS-mEQIP showed good internal consistency and excellent interrater reliability (ICC=0.89, Cronbach's α=0.84), while ChatGPT-mEQIP showed excellent internal consistency and excellent interrater reliability (ICC=0.96, Cronbach's α=0.96).

**Table 3:** Rater scores for "Content" mEQIP items (#1-18) ICC, intraclass correlation coefficient; mEQIP, Modified Ensuring Quality Information for Patients; ASPS, American Society of Plastic Surgery

Characteristics		ASPS	ChatGPT	
Average rater score		0.43	0.94	
ICC		0.89	0.96	
Cronbach's alpha		0.84	0.96	
*ICC: >0.75 (good), >0.9 (excellent)				
*Cronbach's alpha: >0.7 (acceptable), >0.8 (good), >0.9 (excellent)				

## Discussion

In this study, ChatGPT outperformed the ASPS resources in providing patient-level information on gender-affirming top surgery. Using the validated mEQIP tool, ChatGPT demonstrated consistently higher scores compared to the ASPS materials across all domains, with particularly strong performance on content-related items. Paired t-tests showed statistically significant differences favoring ChatGPT, with large effect sizes. These findings suggest that the information generated by ChatGPT may be more comprehensible for a patient or clinician reviewing the material. Additionally, ChatGPT (ICC=0.96, Cronbach’s α=0.96) demonstrated higher consistency and stronger agreement among raters than ASPS (ICC=0.89, Cronbach’s α=0.84).

These findings align with emerging literature evaluating the role of large language models in patient education and surgical communication. Across multiple specialties, including orthopedics, dermatology, ophthalmology, and otolaryngology, ChatGPT has been shown to produce accessible and intelligible educational content compared to traditional online resources [7-19]. This study’s results extend these observations into gender-affirming surgery, a domain where high-quality, patient-centered educational materials remain limited [23-25].

The implications of these findings are noteworthy. With the evolving nature of technology, many patients rely on online resources to inform decision-making prior to consultation, yet the quality of these resources varies widely [26-32]. Thus, ChatGPT’s ability to generate higher-quality explanations may serve as an effective adjunct to existing educational tools in gender-affirming care. This is further supported by prior work noting ChatGPT’s ability to produce quality information in an accessible manner for patients seeking gender-affirming care [33]. Despite its capability to produce relevant content, current patient utilization of ChatGPT for gender-affirming surgery remains limited [34].

Going forward, further development of AI-generated responses may assist patients in better understanding desired procedures, associated risks, and surgical outcomes. With continued tailoring of AI tools to develop health education materials, patients may ultimately possess greater clinical understanding of their gender-affirming care prior to surgical consultation. Of note, AI-generated responses are not intended to replace individualized counseling by a trained surgeon; rather, they may bridge gaps in accessibility, reduce misinformation, and support informed shared decision-making.

Despite these findings, several limitations must be acknowledged. First, this was a single-institution study. Second, a small sample size of four expert raters was used, which may limit generalizability. Third, these findings reflect ChatGPT’s performance at a specific point in its development (ChatGPT-4) and may vary in future evaluations, given the model’s dynamic nature and continual updates. Fourth, because ChatGPT can learn from multiple prompts and follow-up questions, this may lead to varied answers to the same question. To minimize prompt bias, a new account by the lead author was created to mimic a de novo search on information related to gender-affirming top surgery using the adapted "Content"-related mEQIP items. Fifth, for similar reasons, this may limit the reproducibility of answers, and the overall quality of ChatGPT responses may vary. Sixth, this study only compared ChatGPT to one online resource (ASPS). ASPS was chosen because our institution relies heavily on patient education handouts provided by ASPS, and we wanted a direct comparison. Future studies should compare ChatGPT to other available online resources. Finally, although mEQIP provides a validated framework for assessing information quality, it does not capture dimensions such as cultural sensitivity, inclusivity, or personalization, which are critical in gender-affirming care [22]. To address these limitations, future studies should broaden evaluations to include additional surgical procedures, multiple AI models, and comparisons with other professional society resources. Incorporating patient-centered assessments will also be essential to understand how real users perceive, interpret, and apply AI-generated content. Furthermore, longitudinal analyses will help clarify how ChatGPT’s accuracy, readability, and overall utility within the healthcare system change as the model evolves.

## Conclusions

When using the mEQIP tool, ChatGPT-generated patient medical education on gender-affirming top surgery matched or surpassed the current ASPS resources. ChatGPT achieved significantly higher content-specific and overall scores, a larger effect size, and demonstrated greater internal consistency and interrater reliability compared to ASPS. These findings suggest that AI tools, such as ChatGPT, can serve as a valuable adjunct for patient education, offering accessible and high-quality information for patients.

However, AI-generated responses should be viewed as a supplement rather than a substitute for individualized counseling by qualified surgeons. As patient reliance on AI-driven resources continues to grow, it will be essential to ensure that such tools remain accurate, culturally sensitive, and aligned with evolving standards of care. Ongoing evaluation and refinement will help define the role of ChatGPT and similar models in supporting patient education and shared decision-making in plastic and reconstructive surgery.
